# A randomized controlled trial of varenicline and brief behavioral counseling delivered by lay counselors for adolescent vaping cessation: Study protocol

**DOI:** 10.3389/fpsyt.2023.1083791

**Published:** 2023-03-15

**Authors:** Randi M. Schuster, Corinne Cather, Gladys N. Pachas, Lindsay Nielsen, Vanessa Iroegbulem, Jason Dufour, Kevin Potter, Sharon Levy, Kevin M. Gray, A. Eden Evins

**Affiliations:** ^1^Department of Psychiatry, Center of Addiction Medicine, Massachusetts General Hospital, Boston, MA, United States; ^2^Harvard Medical School, Boston, MA, United States; ^3^Adolescent Substance Use and Addiction Program, Boston Children's Hospital, Boston, MA, United States; ^4^Department of Psychiatry and Behavioral Sciences, Medical University of South Carolina, Charleston, SC, United States

**Keywords:** vaping, nicotine, adolescents, cessation, e-cigarette, young adults, varenicline, clinical trial

## Abstract

**Background:**

Approximately one-fifth of high-school seniors and college students currently vape nicotine. Adolescents express a desire to quit vaping, and case reports have shown promise for e-cigarette tapering with dual behavioral and pharmacologic therapies. However, there are no published clinical trials to date that test these intervention approaches for adolescent nicotine vaping cessation. In this three-arm randomized, placebo-controlled, parallel-group study, we aim to assess the efficacy of varenicline in combination with brief behavioral counseling and texting support on vaping cessation in adolescents dependent on vaped nicotine.

**Methods:**

The study will enroll 300 individuals between the ages of 16–25 with daily or near-daily nicotine vaping who reside in the Greater Boston area. Participants will be randomly assigned in a 1:1:1 ratio in blocks of six to one of the three arms: (1) a 12-week course of varenicline titrated to 1 mg bid, brief behavioral counseling delivered by a lay counselor, and an introduction to This is Quitting (TIQ) texting support created by the Truth Initiative; (2) a 12-week course of placebo, brief behavioral counseling, and TIQ; and (3) 12 weeks of enhanced usual care, consisting of advice to quit and an introduction to TIQ. The primary outcome will be biochemically verified continuous vaping abstinence at the end of the treatment (week 12). Secondary outcomes include continuous abstinence at follow-up (week 24), 7-day point prevalence abstinence at weeks 12 and 24, safety and tolerability of varenicline in an adolescent vaping population, as well as change in mood and nicotine withdrawal symptoms across the intervention period. Exploratory outcomes include change in comorbid substance use behaviors and nicotine dependence. Analysis will be intent-to-treat, with multiple imputation sensitivity analyses for participants with missing or incomplete outcome data.

**Discussion:**

This is the first study to evaluate varenicline in combination with a novel, brief, lay counselor delivered vaping cessation program for adolescents who vape nicotine. Results will inform clinicians on the effectiveness and acceptability of this promising, but not yet tested intervention.

**Clinical trial registration**: ClinicalTrials.gov, identifier NCT05367492.

## 1. Introduction

Approximately 20% of US high school seniors and college students report past month nicotine vaping (1, 2), and it is estimated that one million adolescents have become addicted to nicotine *via* vaping (3). Best practice guidelines support discouraging e-cigarette use among adult non-smokers and all adolescents (regardless of smoking status) due to direct health risks of vaping (4), risk for nicotine dependence, and possible increased risk for other substance use disorders (5, 6). Regular vaping is associated with heavy metal and carcinogen exposure (7), wheezing (8), pulmonary inflammation, impaired pulmonary gas exchange (9), and greater morbidity and mortality of COVID-19 (10, 11). Among never-smokers, nicotine vaping is associated with more than 7-fold increased odds of initiating tobacco smoking (12). Nicotine use in youth is associated with cognitive decline, lower psychomotor speed and decreased cognitive flexibility in adulthood (13). There is also growing consensus that vaping nicotine increases the risk of future dependence on cannabis, alcohol, and other substances (14). Vaping cessation is likely to reduce exposure of youth to these and other acute and long-term health risks, even among those who do not smoke tobacco (15, 16).

Adolescents have reported interest in vaping cessation, with health effects noted as a top motivator for quitting (17, 18). Approximately two-thirds of high school students who use e-cigarettes report a vaping cessation attempt in the past year (19–22). The proportion of adolescents attempting to quit vaping doubled between 2018 and 2020 (19, 23), likely driven by implementation of various vaping control policies (e.g., Tobacco 21) and public health campaigns aimed at increasing awareness of potential risks of vaping (24). However, the demand for effective vaping cessation interventions has outpaced science, with no evidence-based treatments currently available for adolescents who vape nicotine and want to quit. Case reports support successful e-cigarette tapering with behavioral and pharmacologic treatment (25, 26).

Varenicline, a selective nicotinic acetylcholine receptor partial antagonist that binds primarily to the α4β2 receptor subtype, may aid vaping cessation by reducing the rewarding effects of nicotine use, nicotine craving, and nicotine withdrawal symptoms (9). Varenicline has consistently been shown to be superior to placebo, nicotine replacement therapy (NRT), and bupropion for smoking cessation in adult populations (27–30), and to be well tolerated in adolescents over the age of 12 (31–34). Randomized controlled trials of varenicline for smoking cessation in adolescents have yielded mixed results (25, 28, 32–37). However, interpretation of these findings is complicated by low medication adherence rates (33, 34). Additionally, less than 3% of US high school students report current tobacco smoking (1), making the adolescent population who smokes tobacco potentially quite different and perhaps more refractory to tobacco use disorder treatment than the population now vaping nicotine.

We will conduct a three-arm randomized controlled trial, comparing 12 weeks of varenicline combined with brief behavioral counseling (V+BC) to identical appearing placebo combined with brief behavioral counseling (P+BC), and enhanced usual care (EUC) involving advice to quit and monitoring. All participants will be encouraged to use ‘This is Quitting’ (TIQ), a free e-cigarette text message quit program developed in 2019 by the Truth Initiative for adolescents and young adults (38).

The primary aim of this study is to evaluate the efficacy of 12 weeks of varenicline, when added to behavioral and texting support, for biochemically verified continuous vaping abstinence at end of treatment among adolescents dependent on vaped nicotine (V+BC vs. P+BC). We will assess the effect of varenicline and behavioral counseling compared to behavioral counseling alone at end of follow-up (week 24) as a secondary outcome. Secondary efficacy outcomes will also include assessing the effect of varenicline and behavioral counseling over enhanced usual care (V+BC vs. EUC) and the effect of behavioral counseling over enhanced usual care (P+BC vs. EUC) on various measures of vaping abstinence.

The secondary aim of this study is to further assess the safety and tolerability of varenicline in adolescents attempting to quit vaping nicotine. For this we postulate that nicotine withdrawal symptoms, craving, and negative mood symptoms will be less frequent and severe in the V+BC than the P+BC group in the treatment period post-quit day, and that the V+BC group will not differ from P+BC and EUC in the incidence and severity of adverse events during the treatment or follow-up periods.

The exploratory aim of this study is to assess if those assigned to V+BC have fewer symptoms of tobacco use disorder and less consumption of alcohol, combusted tobacco, cannabis, and non-medical prescription drugs in the treatment and follow-up periods than those assigned to P+BC or EUC.

## 2. Methods

### 2.1. Design

We will conduct a three-arm randomized, placebo-controlled, parallel-group study comparing 1. a 12-week course of varenicline titrated to 1 mg bid, brief behavioral counseling designed for adolescent vaping cessation, and TIQ texting support (V+BC), 2. placebo, brief behavioral counseling, and TIQ (P+BC), and 3. enhanced usual care (EUC) consisting of brief referral to TIQ texting support and monitoring.

**Figure 1:**
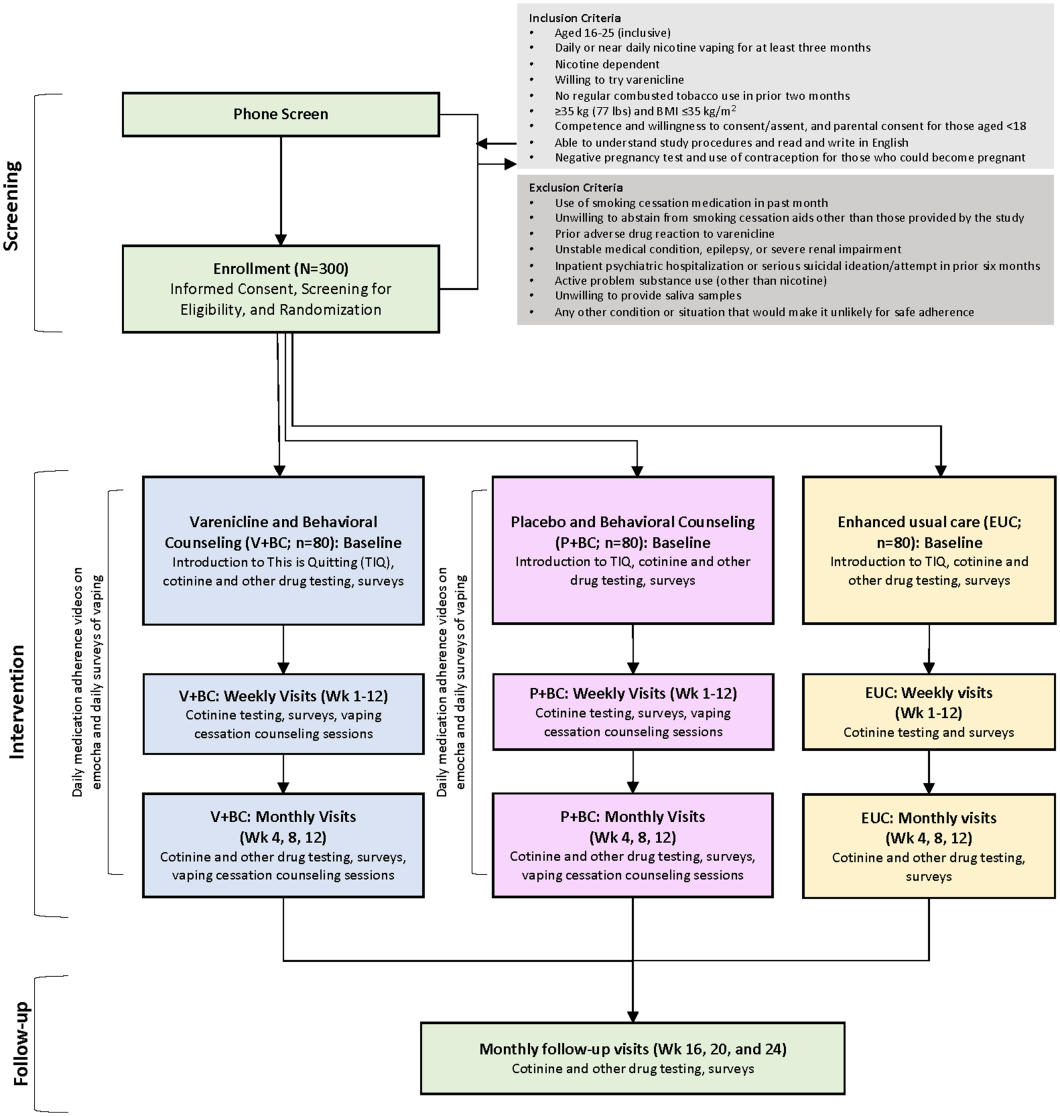
A visual description of study protocol.

All participants will complete weekly self-reported assessments to monitor vaping behavior, receive advice to quit vaping at the first post-baseline weekly visit, and be referred to the TIQ vaping cessation program (Figure 1). Other study-related outcomes will be assessed at the monthly visits (weeks 4, 8, 12, 16, 20, 24; Table 1).

**Table 1:** Time and events.

Assessments	Intervention period							Follow-up period			
	Enrollment	BL/Wk0	Wk 1–3	Wk 4	Wk 5–7	Wk 8	Wk 9–11	Wk 12	Wk 16	Wk 20	Wk 24
Screening											
Demographics	X	–	–	–	–	–	–	–	–	–	–
Vaping history	X	–	–	–	–	–	–	–	–	–	–
Primary outcomes											
Salivary cotinine	X	X	X	X	X	X	X	X	X	X	X
Timeline follow-back	X	X	X*	X	X*	X	X*	X	X	X	X
Daily vaping *via* emocha	–	X	X	X	X	X	X	X	–	–	–
Secondary/exploratory outcomes											
Adverse events, MNWS, QVC	X	X	X	X	X	X	X	X	X	X	X
MASQ-D30, NAEI	X	X	–	X	–	X	–	X	X	X	X
Other biomarkers											
Urine NNAL, THC-COOH	–	X	–	–	–	–	–	X	–	–	X
Pregnancy test	X	X	–	X	–	X	–	X	–	–	–
Urine drug screen	X	X	–	X	–	X	–	X	X	X	X
Vital signs	X	–	–	X	–	X	–	X	–	–	–
Height and weight, CO, genetics	X	–	–	–	–	–	–	–	–	–	–
Descriptions, effect modifiers, covariates											
Medical history	X	–	–	–	–	–	–	–	–	–	–
Concomitant medications	X	X	X	X	X	X	X	X	X	X	X
AUDIT, CUDIT, ECDI	X	–	–	–	–	–	–	X	–	–	X
BRIEF-SR	X	–	–	–	–	–	–	X	–	–	–
C-SSRS, UPPS-P, WTAR	X	–	–	–	–	–	–	–	–	–	–
CHRT	–	X	–	X	–	X	–	X	X	X	X
Flavor preferences, peer and family influence on vaping, vaping and substance use history, modified WTQ, MTQ	X	–	–	–	–	–	–	–	–	–	–
YSS	–	–	–	–	–	–	–	X	–	–	–
Additional study procedures											
TIQ, Quit Vaping Intervention	–	–	X	X	X	X	X	X	–	–	–
Randomization	–	X	–	–	–	–	–	–	–	–	–
Drug distribution	–	X	X**	X	–	X	–	–	–	–	–

*Modified TLFB of past 7-day vaping during weekly visits **Week 2 only.

The trial has been approved by the Mass General Brigham (MGB) Institutional Review Board (IRB). It will be conducted in accordance with the Consolidated Standards for Reporting Trials Statement (39–41), and will be reported here in accordance with the Standard Protocol Items: Recommendations for Intervention Trials statement (42).

### 2.2. Participants

Participants will be included if they are aged 16–25 years (inclusive); report approximately daily nicotine vaping for the prior ≥3 months, bio-verified by saliva cotinine >30 ng/mL; score ≥ 4 on the 10-item E-cigarette Dependence Inventory (ECDI) (43), and/or report persistent use despite negative consequences or prior failed quit attempts; report no regular combusted tobacco use in the prior two months; are ≥35 kg with a body mass index ≤35 kg/m^2^; are competent and willing to consent (age > 18) or assent and have a parent/legal guardian able to consent if under the age 18 years; are able to read and write in English; and have a negative urine pregnancy test and agree to use effective contraception during the study if they are able to become pregnant.

Participants will be excluded who use a smoking cessation medication in the prior month; are unwilling to abstain during the study from using smoking cessation aids other than those provided by the study; experienced a prior adverse drug reaction to varenicline; report an unstable medical condition, epilepsy, or severe renal impairment; had an inpatient psychiatric hospitalization and/or serious suicidal ideation or suicide attempt in the prior 6 months; have active problem substance use (other than nicotine) severe enough to compromise ability to safely participate in the study protocol, in the investigator’s opinion; have any other condition or situation that would, in the investigator’s opinion, make it unlikely that the they could adhere safely to the study protocol.

### 2.3. Sample size

Sample size was determined based on the assumption that use of varenicline for vaping cessation in adolescents would require an effect that at least doubled cessation rates over placebo to be worth the risks inherent in use of pharmacotherapy. Abstinence rates of 24% have been reported with TIQ alone in adolescents who vaped and were not necessarily dependent (44, 45). In this trial, requiring nicotine dependence to enroll, we postulate that addition of varenicline to behavioral counseling and TIQ will double abstinence rates. A sample size of 100 per arm, with 80 analyzable per arm, would yield power of 0.88 with a two-sided alpha of 0.05 with abstinence rates of 25% in the P+BC arm and 50% in the V+BC arm.

Effect estimates were notably not based on reported smoking abstinence rates with varenicline in adolescents dependent on smoked nicotine because these studies yielded low abstinence rates in both the varenicline and placebo groups (33, 34). We speculate that these trials under-estimated the therapeutic effect of varenicline due to modest medication adherence (e.g., 63% took 100% of dispensed doses) and high dropout rates (e.g., 45%) (33). We anticipate abstinence rates will be higher in the current trial due to use of asynchronous video-based directly observed therapy and daily text messages to encourage medication adherence. We also anticipate differences in characteristics among adolescents dependent on smoked vs. vaped nicotine (e.g., number of prior quit attempts, rates of externalizing disorders, concurrent other substance use, level of physical activity) will contribute to higher abstinence rates in all arms of the current trial than observed with prior adolescent smoking cessation trials (46, 47).

### 2.4. Recruitment and retention

Participants will be recruited from local high schools and colleges as well as community and clinical settings. Recruitment of high school-aged students will be augmented through annual high school screenings conducted as part of other ongoing school-based research protocols (PI: Schuster) and direct outreach to school nurses and mental health staff. Eligibility will be assessed through a telephone and/or online survey and confirmed during the enrollment visit. To minimize data loss, we will conduct virtual visits when possible. Retention will be aided by compensation of up to $1,188 per participant for completion of assessments and remotely obsereved adherence to study medcation for V+BC and P+BC groups; see Table 2 for detailed payment schedule. Consent and assent forms will indicate that remuneration will be issued directly to research participants, not parents or caregivers.

**Table 2:** Schedule of remuneration.

	V+BC/P+BC		EUC	
	Per visit	Cumulative amount	Per visit	Cumulative amount
Study Visits				
Enrollment	$20	$20	$20	$20
Baseline (Wk 0)	$25	$45	$25	$45
Week 1	$20	$65	$20	$65
Week 2	$25	$90	$25	$90
Week 3	$30	$120	$30	$120
Week 4+Month 1	$35+$30	$185	$35+$30	$185
Week 5	$40	$225	$40	$225
Week 6	$45	$270	$45	$270
Week 7	$50	$320	$50	$320
Week 8/Month 2	$55+$35	$410	$55+$35	$410
Week 9	$65	$475	$65	$475
Week 10	$75	$550	$75	$550
Week 11	$85	$635	$85	$635
Week 12	$95+$40	$770	$95+$40	$770
Week 16/Month 4 (follow-up 1)	$45	$815	$45	$815
Week 20/Month 5 (follow-up 2)	$50	$865	$50	$865
Week 24/Month 6 (follow-up 3)	$55	$920	$55	$920
Micro-Reimbursements for Adherence				
Medication adherence	$1/day	$84	N/A	N/A
Daily survey of vaping behavior	$1/day	$84	N/A	N/A
Completion Bonus				
Provided >80% of saliva samples		$100		$268
Maximum potential earnings				
		$1188		$1188

### 2.5. Consent or assent

Potentially eligible participants will provide consent (assent if age < 18) following verbal and written explanation of the study, potential risks and voluntary nature of participation, right to withdraw, and details of data protection and confidentiality. Study staff will conduct informed consent procedures with a parent/guardian of participants under the age of 18 prior to initiating study procedures.

### 2.6. Randomization

Eligible participants will be randomly assigned in a 1:1:1 ratio, in blocks of 6, to V+BC, P+BC, or EUC, stratified by secondary or post-secondary school status, according to a randomization scheme computer generated by Massachusetts General Hospital (MGH) Research Pharmacy personnel not otherwise associated with the study. The randomization codes will be held in the MGH research pharmacy and made available to study investigators only in the case of urgent medical need. Enrolled participants will be randomly assigned to a group sequentially based on the date of their baseline visit.

### 2.7. Blinding

Outcome assessors, data managers, and analysts will be blinded to treatment assignments. All study staff and participants will be blind to study medication assignment for the primary outcome comparing V+BC and P+BC. Interventionists and their clinical supervisors will be unblinded to assignment to behavioral counseling and will not be involved in assessments, data management, or data analysis.

### 2.8. Interventions

#### 2.8.1. Enhanced usual care for vaping cessation (EUC)

All study participants will receive brief advice to quit vaping and an introduction to free, anonymous, text-based vaping cessation support through the Truth Initiative (This is Quitting [TIQ])^1^ reported to have efficacy for promoting abstinence in this population (44, 45).

#### 2.8.2. Pharmacotherapy (varenicline or identical placebo)

Participants randomized to V+BC and P+BC will receive generic varenicline or identical appearing placebo. In adolescents aged 12–17 years, steady-state systemic exposure in those who weighed >55 kg was comparable to adults at the same dose; however, steady-state daily exposure of 0.5 mg bid of varenicline was, on average, higher (by approximately 40%) in adolescents who weighed ≤55 kg compared to adults (32). Therefore, for the current study, participants ages 16–17 years old (≤ 55 kg) will take one 0.5 mg dose once daily for the first 7 days before taking one 0.5 mg dose twice daily for 11 weeks. For participants 16–17 years old (>55 kg) and participants 18 years and older, regardless of weight, they will take one 0.5 mg dose once daily for 3 days, one 0.5 mg dose twice daily for 4 days, and then one 1.0 mg dose twice daily for the remaining 11 weeks.

Medications will be adjusted to a lower dose if 1.0 mg bid is not well tolerated. Efficacy of doses lower than 0.5 mg bid have not been reported for any population, thus participants who do not tolerate the dose of varenicline or placebo 0.5 mg bid will be discontinued from medication, but retained in the study and included in the intent-to-treat analyses. Study staff will distribute varenicline or placebo with instructions on how to take the study medication as follows: 2-week supply at baseline, 2-week supply at week 2, and 4-week supplies at weeks 4 and 8.

##### 2.8.2.1. Medication adherence

**Figure 2:**
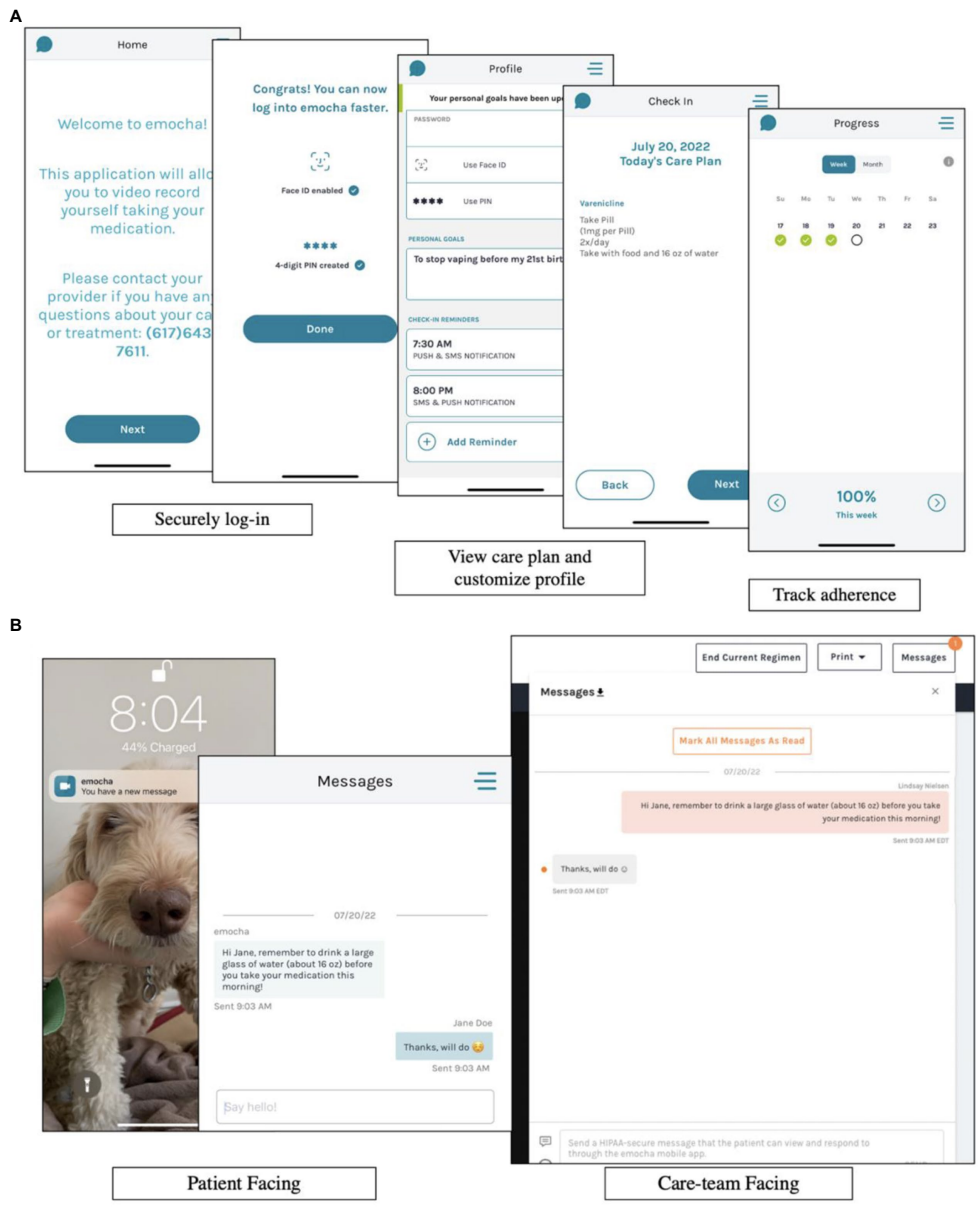
Information regarding emocha, a HIPAA-compliant mobile phone application used for medication adherence. **(A)** Demonstrates how participants are able to securely log in to emocha, view their medication schedule for the day, and track their medication adherence. **(B)** Demonstrates how participants receive phone notifications from emocha, as well as how participants can message study staff securely through the application.

Medication adherence will be assessed during the treatment period with emocha, a HIPAA-compliant mobile phone application that reminds participants *via* phone notifications to take their study medication and assesses adherence with video-based directly observed therapy (vDOT) (see Figure 2). One video will be uploaded for each dose of study medication, and study staff will review video submissions daily to confirm adherence. Recorded dosing will be incentivized with micropayments ($1 per emocha video, up to $2 daily). Reasons for missed doses will be discussed and problem-solved in the behavioral counseling sessions. We will complete a pill count at each visit to supplement adherence data collected through emocha.

#### 2.8.3. Brief behavioral counseling for vaping cessation (Quit Vaping)

Participants randomized to V+BC and P+BC will be enrolled in a novel, 12-week manualized behavioral counseling intervention (Quit Vaping) based on the American Lung Association smoking cessation program (29, 48–50), modified to be adolescent-friendly and relevant to vaping cessation. The intervention contains 12 modules, each of which is designed to be delivered by lay/non-clinically trained personnel in a 20 min session and can be delivered either *via* videoconference or in-person (see Table 3). Content is delivered with psychoeducational videos, guided discussions, and take-home worksheets to consolidate information and provide opportunities for skill building. Example module topics include: identifying pros and cons of vaping, industry tactics, refusal skills, and establishing a healthy lifestyle. All sessions will follow the same general structure, including: 1. check-in on adverse events, vaping behavior, and medication adherence (approximately 5–10 min); 2. presentation of new educational content (*via* pre-recorded psychoeducational videos; approximately 3 min); and 3. review of educational material and relevance to the participant (approximately 7–10 min). Participants will be asked to set a quit date before Session 3 and will work with the counselor during Sessions 1 and 2 to create a detailed quit plan. All session materials will be provided on the intervention website,^2^ along with additional supplementary resources for the participant to access.

**Table 3:** Overview of the brief behavioral counseling intervention for vaping cessation (Quit Vaping).

Session	Name	Content
1	Decisional Balance	Identify personal reasons for vaping and wanting to quit as well as potential factors that reinforce continued use
2	Preparing for Quit Day	Define what triggers are, identify personal triggers, and learn how to avoid/navigate triggers when they occur; Learn how to utilize the 4Ds (delay, drink water, take a deep breath, and distract) to overcome cravings and set a quit day
3 (Quit Day)	Neurobiology of Addiction and Effects of Vaping	Learn about the neurobiology of nicotine addiction, as well as how addiction and nicotine impact the brain and body
4	Slips and Problem Solving	Define what slips are, identify the difference between a slip and a relapse, and identify and practice strategies to avoid or prevent slips from occurring
5	Dealing with Social Pressure While Quitting	Identify social situations where pressure to vape may emerge; Define and practice the REFUSE methods (**R**emind them that you have quit, **E**xplain for make an excuse, **F**ind another focus, **U**se a friend, **S**eparate yourself from the situation, **E**mpower yourself) to help mitigate social pressures to vape while trying to quit
6	Industry Tactics	Identify tactics the vaping industry uses to deceptively advertise and market to adolescents; Consider the ways the vaping industry uses marketing and advertisements to hook young people on vapes and create life-long customers and practice sharing this information to close family members and friends
7	Coping with Stress	Evaluate the relationship between stress and vaping, and practice using alternative ways to cope with stress that do not involve vaping
8	Rewarding Yourself for Your Successes	Identify the importance of rewarding successes, and how setting milestones during the quit journey can also serve as a powerful motivator; Practice setting, tracking, and celebrating milestones throughout the quitting process (e.g., one month quit, three months quit)
9	Staying Motivated	Assess motivation levels at various time points while quitting and identify strategies to boost motivation during inevitable hard times in the quit journey (e.g., tracking the amount of money saved since quitting)
10	Establishing a Healthy Lifestyle	Identify how incorporating healthy choices and habits into one’s day-to-day routine can help maintain one’s quit; Begin to implement other aspects of a healthy lifestyle into daily routine
11	Changes that Come with Quitting	Discuss and reflect on the positive changes as well as challenges people may encounter when quitting vaping (e.g., being more active), and how these may influence a quit journey
12	Planning for Long-Term You	Identify how to plan for the future when it comes to maintaining a long-term quit; Review skills and strategies learned during the program to maintain living a vape-free life

Quit Vaping counselors will complete the Tobacco Treatment Specialist (TTS) training program.^3^ Counselors will also receive 45 min of weekly supervision from a doctoral-level clinical psychologist on study staff for the first six months of the study with monthly supervision thereafter. Supervision will provide opportunities for case consultation as well as information on vaping behavior and cessation in adolescents, study methods, as well as the theoretical rationale and skills relevant to the behavioral intervention.

##### 2.8.3.1. Fidelity monitoring

To monitor fidelity and quality assurance, a staff clinical psychologist will review audio recordings for all 12 sessions for two participants per counselor and provide feedback using a 11-item fidelity scale designed to assess fidelity to session content and non-specific therapy skills. Fidelity assessments will be conducted for 20% of the participants randomized to receive the Quit Vaping intervention and study counselors whose receive three overall fidelity ratings of <3 will be assigned to additional training to improve skills.

### 2.9. Outcome measures

Assessments will occcur at enrollment, baseline (wk0; randomization), weekly for intervention sessions (wk 1–12), and follow-up weeks 16, 20 and 24. Primary, secondary and exploratory outcomes are described below. All measures, including descriptives and potential covariates, are listed in in Table 1.

#### 2.9.1. Primary outcome: Continuous vaping abstinence

The primary outcome is cotinine-verified, continuous nicotine vaping abstinence, operationalized by self-report of no nicotine vaping since the last study visit and salivary cotinine <30 ng/mL (51) at each visit from study week 9 to end of treatment (week 12) (primary) and from study week 9 to the end of follow-up (week 24; secondary). Self-reported vaping will be assessed at all visits using the timeline follow-back (TLFB) (52, 53). A 60-day recall period will be queried at enrollment, and a modified TLFB will be administered at subsequent visits to ascertain vaping in the period between visits. Saliva samples will be collected weekly during the treatment phase and then monthly during follow-up for qualitative cotinine measurement. Study staff will mail collection kits in advance of virtual visits to participants, and participants will provide the sample during the videoconferencing session and show results to the assessor. Those who report no vaping since the last study visit but who have a cotinine >30 ng/mL will be considered non-abstinent at that visit for analyses.

#### 2.9.2. Secondary outcome: Point prevalence vaping abstinence

Using TLFB procedures and salivary cotinine assays, we will assess for differences in 7-day point-prevalence vaping abstinence at end of treatment. Point prevalence abstinence is operationalized as self-report of no vaping in the prior seven days and salivary cotinine <30 ng/mL at week 12.

#### 2.9.3. Secondary outcome: Safety and tolerability of varenicline

We will assess for group differences in frequency and severity of nicotine withdrawal, craving, and negative mood, as well incidence and severity of adverse events. Nicotine withdrawal will be assessed at all study visits with the Minnesota Withdrawal Scale (MNWS) (54), a 9-item scale of nicotine withdrawal symptoms (i.e., craving, irritability, anxiety, difficulty concentrating, restlessness, increased appetite or weight gain, depression, and insomnia) reported on an ordinal scale from 0 (not at all) to 4 (extreme). Craving will be assessed at all study visits using the Questionnaire of Vaping Craving (QVC) (55), a 10-item measure of vaping craving that assesses desire and intent to vape and anticipation of positive outcomes related to e-cigarette use. Negative mood will be assessed at enrollment, baseline and monthly study visits with the Mood and Anxiety Symptoms Questionnaire (MASQ-D30) (56), a 30-item adaptation of the original 96-item MASQ (57, 58), which instructs participants to rate how often in the past week they have experienced symptoms of depression and anxiety on a 5-point Likert scale, with 1 being “not at all” and 5 being “extremely.” Adverse events will be assessed at enrollment, baseline and monthly study visits with the Neuropsychiatric Adverse Events Interview (NAEI) (27), a semi-structured interview designed to assess the presence and severity of symptoms such as irritability and suicidal thoughts or behavior.

#### 2.9.4. Exploratory outcome: Frequency of other substance Use

The TLFB interview will be used to assess frequency and amount of cannabis, alcohol, and illicit substance use 60 days prior to enrollment as well as interim periods at baseline, monthly study visits, and post-intervention follow-ups. Self-report of combustible tobacco use will be biochemically verified with urine NNAL (4- (methylnitrosamino)-1-(3-pyridyl)-1-butanol) at end-of-treatment and follow-up, with <10 pg./mL indicating no combustible tobacco use in the past two months (59). NNAL presence in urine is indicative of exposure to cured tobacco products and as such is not present with vaped nicotine.

### 2.10. Confidentiality

All data collected from participants will be confidential, with the exception being situations of imminent risk to self or others or suspicion of child or elder abuse. Participants younger than age 18 will be told during the telephone screen that the process of seeking consent will inevitably reveal use of nicotine vapes to parents/guardians, and they will be given the opportunity to decline participation at that time. Beyond basic vaping eligibility criteria revealed in consent documents, no information will be shared with parents/guardians of participants under the age of 18 except for in situations as required by law (e.g., acute concern for safety of self or other). Confidentiality will be maintained by numerically coding all data and by keeping all data in password protected, secure, HIPAA compliant databases. All study staff will be trained in protection of privacy of research participants and will have certification from the Collaborative Institutional Training Initiative. This study will maintain a Certificate of Confidentiality from the National Institutes of Health to protect against forced disclosure of identifiable, sensitive information collected as part of this clinical trial.

### 2.11. Data analysis

#### 2.11.1. Missing data

Missing or incomplete outcome data from study week 9 to 12 will be imputed 40 times using multiple imputation *via* chained equations (60). Outcome data will be imputed using three predictors collected at the enrollment visit: biological sex (male versus female), environment (secondary school versus post-secondary school), and severity of nicotine dependence (ECDI, summed score ranging from 0–20) (61). Imputation will be done *via* fully conditional specification with predictive mean matching using linear models for continuous data and logistic regression for binary variables.

#### 2.11.2. Analytic plan for abstinence outcomes

We will use a logistic regression applied to the binary outcome for 4-week continuous abstinence at end of treatment (weeks 9–12; primary) and end of follow-up (weeks 9–24) (1 = abstinent, 0 = not abstinent). Participants whose observed continuous abstinence status cannot be determined due to missing data will have continuous abstinence status determined *via* imputation. We will include sex, environment (secondary school versus post-secondary school), and severity of nicotine dependence (ECDI) as baseline covariates which will be standardized for analyses. The analysis will be intent-to-treat. We will then apply our logistic regression model to each set of observed and imputed data, and pool the regression estimates according to Rubin’s rule. Statistical significance for the contrast between V+BC and P+BC will be determined using a *z*-test applied to the pooled mean estimate divided by the pooled standard error. The same analyses will be applied for secondary hypotheses comparing V+BC and P+BC vs. EUC on 4-week continuous abstinence at end of treatment and follow-up as well as all groups on seven-day point-prevalence vaping abstinence at end of treatment and follow-up.

#### 2.11.3. Analytic plan for withdrawal, craving, and mood outcomes

We will use a linear model applied separately to repeated, continuous weekly assessments of nicotine withdrawal symptoms, craving, and mood symptoms. The linear model will be estimated using generalized estimating equations, providing estimates and standard errors robust to violations of distributional assumptions (e.g., normality) and heteroscedasticity. The model will be applied to the summed scores at each time point per participant. The analysis will be intent-to-treat with missing data imputed. The key confirmatory effect will be a dummy-coded contrast comparing placebo (P+BC; coded as 0) against varenicline (V+BC; coded as 1). Exploratory analyses will also be conducted to evaluate V+BC vs. EUC and P+BC vs. EUC. The model will include covariates for change over time (two terms encoding linear and quadratic trends), baseline levels of the outcome variable, and sex, environment (secondary school versus post-secondary school), and severity of nicotine dependence. Covariates will be standardized. *p*-values will be adjusted to avoid multiple comparison issues using the Benjamini-Hochberg method.

#### 2.11.4. Analytic plan for adverse events outcome

We will conduct a Fisher’s exact test to evaluate differences between the V+BC and P+BC groups and V+BC and EUC groups in the number (%) of participants who endorse an adverse event at least once in the 12-week treatment period. We will report frequent adverse events (e.g., that occur in at least 5% of the participants at any point between weeks 1 and 12 of the trial) by group.

#### 2.11.5. Sensitivity analyses

We will test whether our conclusion on efficacy is robust to the inclusion/exclusion of the three covariates [biological sex, environment (secondary school vs. post-secondary school), and nicotine dependence severity]. To test whether our conclusion on efficacy is robust to the approach for imputation of missing data, we will rerun the adjusted (i.e., with covariates) models on the observed data only and will rerun the adjusted models on a dataset with the observed data and all missing outcome data imputed as non-abstinent. Finally, we will conduct a per protocol analysis by evaluating results among participants who use varenicline on most days in the trial (e.g., >50 or 75%).

#### 2.11.6. Software

All analyses will be conducted using the statistical software R (version 4.1.1) (62). Multiple imputation will be carried out using the R package ‘mice’ (version 3.13.0) (60). The generalized estimating equations method will be implemented *via* the R package ‘geepack’ (version 1.3-2) (63).

### 2.12. Safety monitoring

Adverse events will be collected and reported as per Good Clinical Practice guidelines. All adverse events volunteered, observed, or solicited will be recorded and will include the dates of occurrence, severity, assessment of relationship to study drug, countermeasure(s), outcome, and date of resolution for the duration of the study. The principal investigators (Evins and Schuster) will meet weekly with study investigators to review the details of recruitment, data acquisition, and occurrence of adverse events of moderate severity or greater.

An independent Data and Safety Monitoring Board (DSMB) will be appointed for this study. The DSMB will meet to analyze interim results to assess the safety of the study drug at frequent intervals for the duration of the study by determining whether an increased number of adverse events occur among study participants receiving drug compared to participants receiving placebo. The DSMB will include a statistician, an adolescent substance use expert, a pediatrician, and a psychiatrist. Each member of the board will not otherwise be associated with the trial. The DSMB will receive all communication with the MGB IRB and with the Food and Drug Administration as well as summary reports on recruitment, retention and description of all adverse events for review at regular meetings. Serious adverse events and other significant safety issues will be reported immediately to the DSMB and to the IRB per institutional and funding guidelines. Participant information provided to the board and communicated to regulatory authorities *via* summary reports will be identified only with study identifiers to protect confidentiality.

## 3. Discussion

There are few published studies of behavioral or pharmacologic approaches for adolescent vaping cessation (45, 64, 65). Because varenicline has been found to be consistently more effective than other pharmacotherapies for smoking cessation in adults and has demonstrated encouraging secondary abstinence outcomes in two adolescent smoking cessation randomized controlled trials (33, 34), we will conduct a rigorous three-armed randomized controlled trial in nicotine dependent adolescents of biochemically validated vaping abstinence with varenicline, combined with brief, lay counselor delivered behavioral counseling and texting support (27, 29, 30, 33). The two prior trials of varenicline for adolescent smoking cessation have had challenges with medication adherence and retention. The current trial will use an asynchronous video-based directly observed therapy platform^4^ to improve treatment adherence and study retention.

This will be the first study of its kind to rigorously evaluate a promising but not yet tested cessation intervention for the growing number of adolescents addicted to vaped nicotine. For the current trial, we are principally interested in determining the effect of varenicline combined with brief behavioral counseling compared to placebo with behavioral counseling. We hypothesize varenicline, when delivered in combination with behavioral counseling and freely available text-based support, will improve abstinence outcomes compared to behavioral counseling or text-based support alone. We also have an *a priori* plan to evaluate the effect of varenicline paired with the behavioral counseling intervention compared to text-based support as well as the effect of placebo combined with the behavioral counseling intervention compared to text-based support. Future studies will be needed to assess the effect of varenicline when added to the TIQ app alone, which is currently the most likely available treatment for most adolescents.

## 4. Ethics and dissemination

All procedures involved in this trial are consistent with Good Clinical Practice according to the Declaration of Helsinki as well as other generally accepted standards of ethical practice. All procedures were reviewed and approved by the IRB, and this trial is registered on ClinicalTrials.gov (NCT05367492). Any protocol changes will be submitted in an amendment to the IRB for approval, with significant changes updated on ClinicalTrials.gov and reflected in the final outcomes paper. Upon conclusion of the trial, results will also be reported on ClinicalTrials.gov.

Study investigators are committed to the open, timely, and efficient dissemination of research outcomes. All members of the investigative team agree to adhere to the National Institute of Health (NIH) Principles and Guidelines for Recipients of NIH Research Grants and Contracts on Obtaining and Disseminating Biomedical Research Programs. All manuscripts published from these data will be submitted to PubMed Central. A final de-identified data set produced by this research will be stored in a searchable repository, available on request with IRB approval, for retrieval and analysis.

## Ethics statement

The studies involving human participants were reviewed and approved by Mass General Brigham. Participants between the ages of 18-25 years will provide their written informed consent to participate in this study. For participants under the age of 18 years, written informed consent to participate in this study will be provided by the participants’ legal guardian/next of kin.

## Author contributions

RS and AE conceived and designed the study. GP, LN, VI, and JD made relevant contributions to the study design and procedure. CC was the lead developer of the behavioral counseling intervention and fidelity monitoring protocol. KP developed the statistical analysis plan with input from RS and AE. RS wrote the first draft of the paper. All authors read the manuscript, made significant contributions, and approved the final version.

## Funding

Funds for this study are provided by National Institute on Drug Abuse (NIDA; R01DA052583). The funder had no role in study design, decision to publish, or preparation of the manuscript.

## Conflict of interest

SL serves as an expert witness in the case against JUUL. KG has provided consultation to Pfizer, Inc., and Jazz Pharmaceuticals. AE receives NIDA Grant subcontracts from Brain Solutions, and Charles River Analytics, is on the Data Safety and Monitoring Board for Karuna Pharmaceuticals, and performs Advisory Board work for Alkermes.

The remaining authors declare that the research was conducted in the absence of any commercial or financial relationships that could be construed as a potential conflict of interest.

## Publisher’s note

All claims expressed in this article are solely those of the authors and do not necessarily represent those of their affiliated organizations, or those of the publisher, the editors and the reviewers. Any product that may be evaluated in this article, or claim that may be made by its manufacturer, is not guaranteed or endorsed by the publisher.

## Footnotes

^1^https://truthinitiative.org/thisisquitting

^2^https://quitvaping.mgh.harvard.edu/

^3^https://www.umassmed.edu/tobacco/training/ttscore/

^4^https://emocha.com/

## Abbreviations

AUDIT: Alcohol Use Disorders Identification Test
BL: Baseline
BRIEF-SR: Behavior Rating Inventory of Executive Function—Self-Report
CHRT: Concise Health Risk Tracking Instrument
CO: Carbon monoxide
C-SSRS: Columbia-Suicide Severity Rating Scale Screener
CUDIT: Cannabis Use Disorders Test
DSMB: Data and Safety Monitoring Board
ECDI: E-Cigarette Dependence Inventory
EUC: enhanced usual care
IRB: Instritutional Review Board
MASQ-D30: Mood and Anxiety Symptoms Questionnaire
MGB: Mass General Brigham
MGH: Massachusetts General Hospital
MNWS: Minnesota Nicotine Withdrawal Scale
MTQ: Motivation to Quit
NAEI: Neuropsychiatric Adverse Events Interview
NIDA: National Institute on Drug Abuse
NIH: National Institute of Health
NNAL: 4-(methylnitrosamino)-1-(3-pyridyl)-1-butanol
NRT: nicotine replacement therapy
PATH: Population Assessment of Tobacco and Health
P+BC: identical appearing placebo with brief beahvioral counseling
QVC: The Questionnaire of Vaping Craving
THC-COOH: tetrahydrocannabinol carboxylic acid
TIQ: This is Quitting
TLFB: timeline follow-back
UPPS-P: Impulsive Behavior Scale – Short
V+BC: varenicline combined with brief behavioral counseling
Wk: week
WTAR: Welscher Test of Adult Reading
WTQ: Willingness to Quit
YSS: Youth Satisfaction Survey
